# Supplementary material for: What do the sustainable development goals reveal, and are they sufficient for sustainable development?
Source: PLoS One. 2024 Nov 4;19(11):e0310089. doi: 10.1371/journal.pone.0310089 (PMC11534252; doi:10.1371/journal.pone.0310089)
Supplement: S1 Appendix — (DOCX) [file pone.0310089.s001.docx]

**S1 Appendix. Criteria and screening steps used to select indicators and countries.**

As for the indicator selection, we followed the criteria below:

1. Indicators should cover sufficient countries rather than those with sufficient data in only a few countries (i.e., developed countries).
2. Indicators should be expressed in the form of continuous numerical variables. Dichotomous variables (0 or 1) and ordinal variables (1-5: unsatisfied to satisfied) are excluded.
3. Total indicators rather than sub-indicators (i.e., those classified by sex and location) were chosen as far as possible.
4. Indicators that can be compared globally were selected rather than those just for a subset of specific regions or categories of countries. For example, indicators in SDG 14 (Life Below Water) and other indicators related to oceans, inner lakes and mountains are not considered.
5. Ratio, rate, or per capita indicators are selected as far as possible to minimize the impact of the size and volume of the country, such as 12.2.2 (domestic material consumption per GDP), 13.2.2 (CO2 emissions metric tons per capita), IG 3 (ecological footprint per capita).
6. As for the real GDP growth rate indicators (8.1.1 and 8.2.1), arithmetic means between 2015 and 2019 (5 years) were adopted to reduce the impact of the abnormal value of the single year (2019) on the statistical results.
7. On the premise of using indicators from the official database of the United Nations as many as possible, this study replaced a few indicators (i.e., 2.2.1, 4.2.2, 8.5.2, 11.1.1) that do not meet the first criteria with similar ones from the World Bank WDI database. Generally, the proportion of indicators from the UN Global SDG database reached 87%.
8. Illustrative groups (IGs) 1-4 have not participated in the MFA and HCPC. IG 1 contains GDPP (GDP per capita), IG 2 includes HDI, IG 3 comprises EPI, and IG 4 is made up of HPI. Significantly, these four groups were selected for comparison purposes with the SDGs.

After the above selection, 215 countries (and regions) and 95 indicators (excluding four illustrative indicators) are screened. Then, the remaining indicators and countries underwent the second screening, followed by the missing data imputation. Criteria and steps are presented below:

1. For indicators with no 2019 data, the data of the nearest year were used instead (1.3.1, 3.1.1, 3.d.1, 5.2.1, 6.5.1, 8.10.2, 11.1.1, 16.7.1, EFP).
2. Based on the first step, delete countries with a proportion of missing values greater than 30%, leaving 164 countries.
3. Based on the second step, delete indicators with a proportion of missing values greater than 30%, leaving 78 indicators.
4. Based on the third step, we filled in missing values via the classic LOCF (Last observation forward) method with the most recent data from 2014 to 2018 (within five years).
5. Country samples whose missing values were still greater than 7% after filling have been deleted. Regarding remaining samples, we further filled in their missing values by searching data from other official databases (i.e., UNESCO), international economic and political organizations (i.e., OECD, EU), UN reports, and the mean values of region or income.

Finally, we managed to keep 133 countries (S3 Table) and 78 indicators (exclude four illustrative indicators) (S1 Table) that were proceeded for MFA and HCPC.
