# Supplementary material for: What do the sustainable development goals reveal, and are they sufficient for sustainable development?
Source: PLoS One. 2024 Nov 4;19(11):e0310089. doi: 10.1371/journal.pone.0310089 (PMC11534252; doi:10.1371/journal.pone.0310089)
Supplement: S1 Table — (DOCX) [file pone.0310089.s002.docx]

**S1 Table. List of selected SDGs and illustrative groups.**

| **Indicator** | **Tier Category** | **Type** | **Source** | **Year** |
| --- | --- | --- | --- | --- |
| **SDG 1: No Poverty** | | | | |
| 1.1.1(a): Proportion of population below international poverty line (%) | I | N | UNSDG | 2019 |
| 1.1.1(b): Proportion of the employed population below international poverty line (%) | I | N | UNSDG | 2019 |
| 1.3.1: Proportion of population covered by at least one social protection benefit (%) | I | P | UNSDG | 2020 |
| 1.4.1(a): Proportion of population using basic drinking water services (%) | I | P | UNSDG | 2019 |
| 1.4.1(b): Proportion of population using basic sanitation services (%) | I | P | UNSDG | 2019 |
| **SDG 2:** **Zero hunger** | | | | |
| 2.1.1: Prevalence of undernourishment (%) | I | N | UNSDG | 2019 |
| 2.2.1: Prevalence of stunting, height for age (modeled estimate, % of children under 5) | I | N | WDI | 2019 |
| 2.2.3: Proportion of women aged 15-49 years with anaemia (%) | I | N | UNSDG | 2019 |
| 2.a.1: Agriculture orientation index for government expenditures | I | P | UNSDG | 2019 |
| 2.c.1: Indicator of Food Price Anomalies (IFPA), by Consumer Food Price Index | I | N | UNSDG | 2019 |
| **SDG 3: Good health and well-being** | | | | |
| 3.1.1: Maternal mortality ratio | I | N | UNSDG | 2017 |
| 3.2.1: Under-five mortality rate (deaths per 1,000 live births) | I | N | UNSDG | 2019 |
| 3.2.2: Neonatal mortality rate (deaths per 1,000 live births) | I | N | UNSDG | 2019 |
| 3.3.2: Tuberculosis incidence (per 100,000 population) | I | N | UNSDG | 2019 |
| 3.4.1: Mortality rate attributed to cardiovascular disease, cancer, diabetes or chronic respiratory disease (probability) | I | N | UNSDG | 2019 |
| 3.4.2: Suicide mortality rate (deaths per 100,000 population) | I | N | UNSDG | 2019 |
| 3.5.2: Alcohol consumption per capita (aged 15 years and older) within a calendar year (litres of pure alcohol) | I | N | UNSDG | 2019 |
| 3.6.1: Death rate due to road traffic injuries (per 100,000 population) | I | N | UNSDG | 2019 |
| 3.7.2: Adolescent fertility rate (births per 1,000 women ages 15-19) | I | N | WDI | 2019 |
| 3.8.1: Universal health coverage (UHC) service coverage index | I | P | UNSDG | 2019 |
| 3.9.1: Age-standardized mortality rate attributed to household and ambient air pollution (deaths per 100,000 population) | I | N | UNSDG | 2019 |
| 3.9.2: Mortality rate attributed to unsafe water, unsafe sanitation and lack of hygiene from diarrhoea, intestinal nematode infections, malnutrition and acute respiratory infections (deaths per 100,000 population) | I | N | UNSDG | 2019 |
| 3.9.3: Mortality rate attributed to unintentional poisonings (deaths per 100,000 population) | I | N | UNSDG | 2019 |
| 3.a.1: Age-standardized prevalence of current tobacco use among persons aged 15 years and older (%) | I | N | UNSDG | 2019 |
| 3.b.1(a): Proportion of the target population with access to 3 doses of diphtheria-tetanus-pertussis (DTP3) (%) | I | P | UNSDG | 2019 |
| 3.b.1(b): Proportion of the target population with access to measles-containing-vaccine second-dose (MCV2) (%) | I | P | UNSDG | 2019 |
| 3.c.1: Health worker density, nursing and midwifery personnel (per 10,000 population) | I | P | UNSDG | 2019 |
| 3.d.1: Average of the International Health Regulations (IHR) capacity (01-13) (%) | I | P | UNSDG | 2017 |
| **SDG 4:** **Quality education** | | | | |
| 4.1.2: Completion rate, lower secondary education, both sexes (%) | I | P | UNSDG | 2019 |
| 4.2.2: School enrollment, preprimary (% gross) | I | P | WDI | 2019 |
| 4.5.1: School enrollment, tertiary (gross), gender parity index (GPI) | I | P | WDI | 2019 |
| **SDG 5:** **Gender equality** | | | | |
| 5.2.1: Proportion of ever-partnered women and girls subjected to physical and/or sexual violence by a current or former intimate partner in the previous 12 months (%) | I | N | UNSDG | 2018 |
| 5.5.1: Proportion of seats held by women in national parliaments (% of total number of seats) | I | P | UNSDG | 2019 |
| 5.5.2: Proportion of women in managerial positions (%) | I | P | UNSDG | 2019 |
| **SDG 6:** **Clean water and sanitation** | | | | |
| 6.4.2: Level of water stress: freshwater withdrawal as a proportion of available freshwater resources (%) | I | N | UNSDG | 2019 |
| 6.5.1: Degree of integrated water resources management implementation (%) | I | P | UNSDG | 2020 |
| 6.6.1: Lakes and rivers permanent water area change (%) | I | P | UNSDG | 2019 |
| **SDG 7:** **Affordable and clean energy** | | | | |
| 7.1.1: Proportion of population with access to electricity (%) | I | P | UNSDG | 2019 |
| 7.1.2: Proportion of population with primary reliance on clean fuels and technology (%) | I | P | UNSDG | 2019 |
| 7.2.1: Renewable energy share in the total final energy consumption (%) | I | P | UNSDG | 2019 |
| 7.3.1: Energy intensity level of primary energy (megajoules per constant 2017 purchasing power parity GDP) | I | N | UNSDG | 2019 |
| **SDG 8:** **Decent work and economic growth** | | | | |
| 8.1.1: Average of the annual growth rate of real GDP per capita (%) between 2015 and 2019 | I | P | UNSDG | 2019 |
| 8.2.1: Average of the annual growth rate of real GDP per employed person (%) between 2015 and 2019 | I | P | UNSDG | 2019 |
| 8.5.2: Unemployment, total (% of total labor force) (modeled ILO estimate) | I | N | WDI | 2019 |
| 8.10.1: Number of commercial bank branches per 100,000 adults | I | P | UNSDG | 2019 |
| 8.10.2: Proportion of adults (15 years and older) with an account at a financial institution or mobile-money-service provider (% of adults aged 15 years and older) | I | P | UNSDG | 2017 |
| **SDG 9:** **Industry, innovation and infrastructure** | | | | |
| 9.1.2: Air transport, passengers carried | I | P | WDI | 2019 |
| 9.2.1: Manufacturing value added (constant 2015 United States dollars) as a proportion of GDP (%) | I | P | UNSDG | 2019 |
| 9.4.1: Carbon dioxide emissions per unit of manufacturing value added (kilogrammes of CO2 per constant 2015 United States dollars) | I | N | UNSDG | 2019 |
| 9.5.1: Research and development expenditure as a proportion of GDP (%) | I | P | UNSDG | 2019 |
| 9.b.1: Proportion of medium and high-tech manufacturing value added in total value added (%) | I | P | UNSDG | 2019 |
| 9.c.1: Proportion of population covered by at least a 2G mobile network (%) | I | P | UNSDG | 2019 |
| **SDG 10:** **Reduced inequalities** | | | | |
| 10.4.1: Labour share of GDP (%) | I | P | UNSDG | 2019 |
| 10.4.2: Gini index | II | N | WDI | 2019 |
| 10.7.4: Number of refugees per 100,000 population, by country of origin (per 100,000 population) | I | N | UNSDG | 2019 |
| 10.a.1: Proportion of tariff lines applied to imports with zero-tariff (%) | I | P | UNSDG | 2019 |
| **SDG 11:** **Sustainable cities and communities** | | | | |
| 11.1.1: Population living in slums (% of urban population) | I | N | WDI | 2018 |
| 11.5.1: Number of deaths and missing persons attributed to disasters per 100,000 population (number) | I | N | UNSDG | 2019 |
| 11.6.2: Annual mean levels of fine particulate matter (population-weighted) (micrograms per cubic meter) | I | N | UNSDG | 2019 |
| **SDG 12:** **Responsible consumption and production** | | | | |
| 12.2.2: Domestic material consumption per unit of GDP (kilograms per constant 2015 United States dollars) | I | N | UNSDG | 2019 |
| 12.3.1: Average food waste per capita (KG) of households, out-of-home consumption and retail | II | N | UNSDG | 2019 |
| 12.b.1: Implementation of standard accounting tools to monitor the economic and environmental aspects of tourism (number of tables) | I | P | UNSDG | 2019 |
| 12.c.1: Fossil-fuel subsidies (consumption and production) as a proportion of total GDP (%) | I | N | UNSDG | 2019 |
| **SDG 13:** **Climate action** | | | | |
| 13.2.2: CO2 emissions (metric tons per capita) | I | N | WDI | 2019 |
| **SDG 15:** **Life on land** | | | | |
| 15.1.1: Forest area as a proportion of total land area (%) | I | P | UNSDG | 2019 |
| 15.1.2: Average proportion of Terrestrial Key Biodiversity Areas (KBAs) covered by protected areas (%) | I | P | UNSDG | 2019 |
| 15.2.1: Annual forest area change rate (%) | I | P | UNSDG | 2019 |
| 15.5.1: Red List Index | I | P | UNSDG | 2019 |
| **SDG 16:** **Peace, justice and strong institutions** | | | | |
| 16.1.1: Number of victims of intentional homicide per 100,000 population | I | N | UNSDG | 2019 |
| 16.3.2: Unsentenced detainees as a proportion of overall prison population (%) | I | N | UNSDG | 2019 |
| 16.7.1: Ratio for female members of parliaments (Ratio of the proportion of women in parliament in the proportion of women in the national population with the age of eligibility as a lower bound boundary), Lower Chamber or Unicameral | I | P | UNSDG | 2021 |
| 16.9.1: Completeness of birth registration (%) | I | P | WDI | 2019 |
| **SDG 17:** **Partnership for the Goals** | | | | |
| 17.1.1: Total government revenue (budgetary central government) as a proportion of GDP (%) | I | P | UNSDG | 2019 |
| 17.1.2: Proportion of domestic budget funded by domestic taxes (% of GDP) | I | P | UNSDG | 2019 |
| 17.3.1: Foreign direct investment (FDI) inflows (millions of US dollars) | I | P | UNSDG | 2019 |
| 17.6.1: Fixed Internet broadband subscriptions (per 100 inhabitants) | I | P | UNSDG | 2019 |
| 17.8.1: Internet users per 100 inhabitants | I | P | UNSDG | 2019 |
| 17.10.1: Worldwide weighted tariff-average, most-favoured-nation status (%) | I | N | UNSDG | 2019 |
| **IG 1: Economic growth** | | | | |
| GDP per capita (current US$) (GDPP) |  | P | WDI | 2019 |
| **IG 2: Prominent human development measure** | | | | |
| Human Development Index (HDI) |  | P | UNDP | 2019 |
| **IG 3: Environmental pressure** | | | | |
| Ecological Footprint (gha per person) (EFP) |  | N | GFN | 2018 |
| **IG 4: Environmental pressure and well-being** | | | | |
| Happy Planet Index (HPI) |  | P | NEF | 2019 |

Note: “P” represents positive indicators, while “N” denotes negative ones.
