# Supplementary material for: What do the sustainable development goals reveal, and are they sufficient for sustainable development?
Source: PLoS One. 2024 Nov 4;19(11):e0310089. doi: 10.1371/journal.pone.0310089 (PMC11534252; doi:10.1371/journal.pone.0310089)
Supplement: S2 Table — (DOCX) [file pone.0310089.s003.docx]

**S2 Table. Correlations between SDGs, IGs and first four PCs.**

|  | PC 1 | PC 2 | PC 3 | PC 4 |
| --- | --- | --- | --- | --- |
| SDG 1 | 0.92 | 0.31 | 0.33 | 0.18 |
| SDG 2 | 0.94 | 0.31 | 0.15 | 0.40 |
| SDG 3 | 0.95 | 0.53 | 0.45 | 0.29 |
| SDG 4 | 0.89 | 0.43 | 0.37 | 0.14 |
| SDG 5 | 0.79 | 0.61 | 0.54 | 0.42 |
| SDG 6 | 0.55 | 0.60 | 0.49 | 0.32 |
| SDG 7 | 0.84 | 0.38 | 0.28 | 0.42 |
| SDG 8 | 0.80 | 0.28 | 0.31 | 0.83 |
| SDG 9 | 0.76 | 0.60 | 0.31 | 0.34 |
| SDG 10 | 0.75 | 0.69 | 0.48 | 0.45 |
| SDG 11 | 0.83 | 0.56 | 0.17 | 0.14 |
| SDG 12 | 0.74 | 0.59 | 0.20 | 0.30 |
| SDG 13 | 0.74 | 0.18 | 0.05 | 0.21 |
| SDG 15 | 0.40 | 0.62 | 0.51 | 0.38 |
| SDG 16 | 0.77 | 0.44 | 0.76 | 0.43 |
| SDG 17 | 0.93 | 0.45 | 0.35 | 0.37 |
| IG 1 (GDPP) | 0.74 | 0.19 | 0.30 | -0.09 |
| IG 2 (HDI) | 0.97 | -0.04 | -0.05 | -0.04 |
| IG 3 (EFP) | -0.78 | -0.09 | -0.05 | 0.09 |
| IG 4 (HPI) | 0.33 | 0.08 | -0.25 | 0.14 |

Note: IG 1-4 are illustrative groups not involved in the MFA and HCPC.
