# Supplementary material for: What do the sustainable development goals reveal, and are they sufficient for sustainable development?
Source: PLoS One. 2024 Nov 4;19(11):e0310089. doi: 10.1371/journal.pone.0310089 (PMC11534252; doi:10.1371/journal.pone.0310089)
Supplement: S3 Table — (DOCX) [file pone.0310089.s004.docx]

**S3 Table. Clusters derived from HCPC.**

| Cluster 1 | Cluster 2 | | Cluster 3 |
| --- | --- | --- | --- |
| Afghanistan | Albania | Morocco | Australia |
| Angola | Algeria | Nicaragua | Austria |
| Bangladesh | Argentina | Panama | Belarus |
| Burkina Faso | Armenia | Paraguay | Belgium |
| Burundi | Azerbaijan | Peru | Bulgaria |
| Cambodia | Belize | Philippines | Canada |
| Cameroon | Bhutan | Samoa | China |
| Congo | Bolivia | Saudi Arabia | Denmark |
| Cote d’Ivoire | Bosnia and Herzegovina | South Africa | Estonia |
| Eswatini | Botswana | Sri Lanka | Finland |
| Ethiopia | Brazil | Suriname | France |
| Gambia | Cabo Verde | Tajikistan | Germany |
| Ghana | Chile | Thailand | Greece |
| Guinea | Colombia | Trinidad and Tobago | Hungary |
| Kenya | Costa Rica | Tunisia | Iceland |
| Lao PDR | Dominican Republic | Türkiye | Israel |
| Lesotho | Ecuador | Ukraine | Italy |
| Madagascar | Egypt | Uruguay | Japan |
| Malawi | El Salvador | Uzbekistan | Korea, Rep. |
| Mali | Fiji | Vietnam | Latvia |
| Mozambique | Georgia |  | Lithuania |
| Myanmar | Guatemala |  | Malta |
| Namibia | Honduras |  | Netherlands |
| Nepal | India |  | New Zealand |
| Niger | Indonesia |  | Norway |
| Nigeria | Iran, Islamic Rep. |  | Poland |
| Pakistan | Iraq |  | Portugal |
| Papua New Guinea | Jamaica |  | Romania |
| Rwanda | Jordan |  | Russian Federation |
| Sao Tome and Principe | Kazakhstan |  | Serbia |
| Senegal | Kyrgyzstan |  | Singapore |
| Solomon Islands | Lebanon |  | Slovenia |
| Sudan | Malaysia |  | Spain |
| Tanzania | Mauritius |  | Sweden |
| Togo | Mexico |  | Switzerland |
| Uganda | Moldova |  | United Kingdom |
| Zambia | Mongolia |  | United States |
| Zimbabwe | Montenegro |  |  |
| GDPP average:  1526.04  HDI average:  0.54 | GDPP average:  6580.72  HDI average:  0.75 | | GDPP average:  37194.14  HDI average:  0.90 |
