# Supplementary figures and images for: What do the sustainable development goals reveal, and are they sufficient for sustainable development?
Source: PLoS One. 2024 Nov 4;19(11):e0310089. doi: 10.1371/journal.pone.0310089 (PMC11534252; doi:10.1371/journal.pone.0310089)

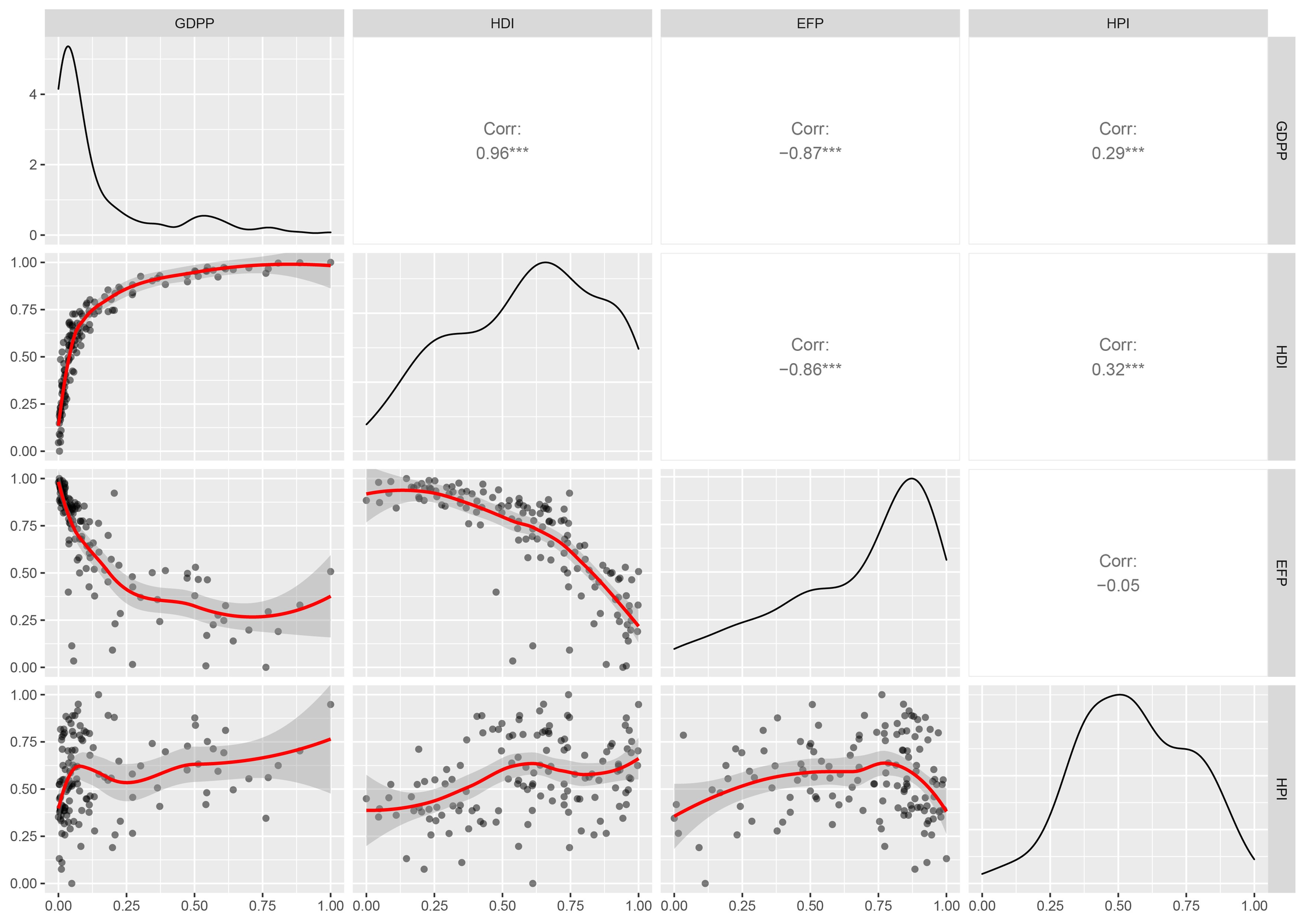

Supplement: S1 Fig — (TIF) [file pone.0310089.s005.tif]

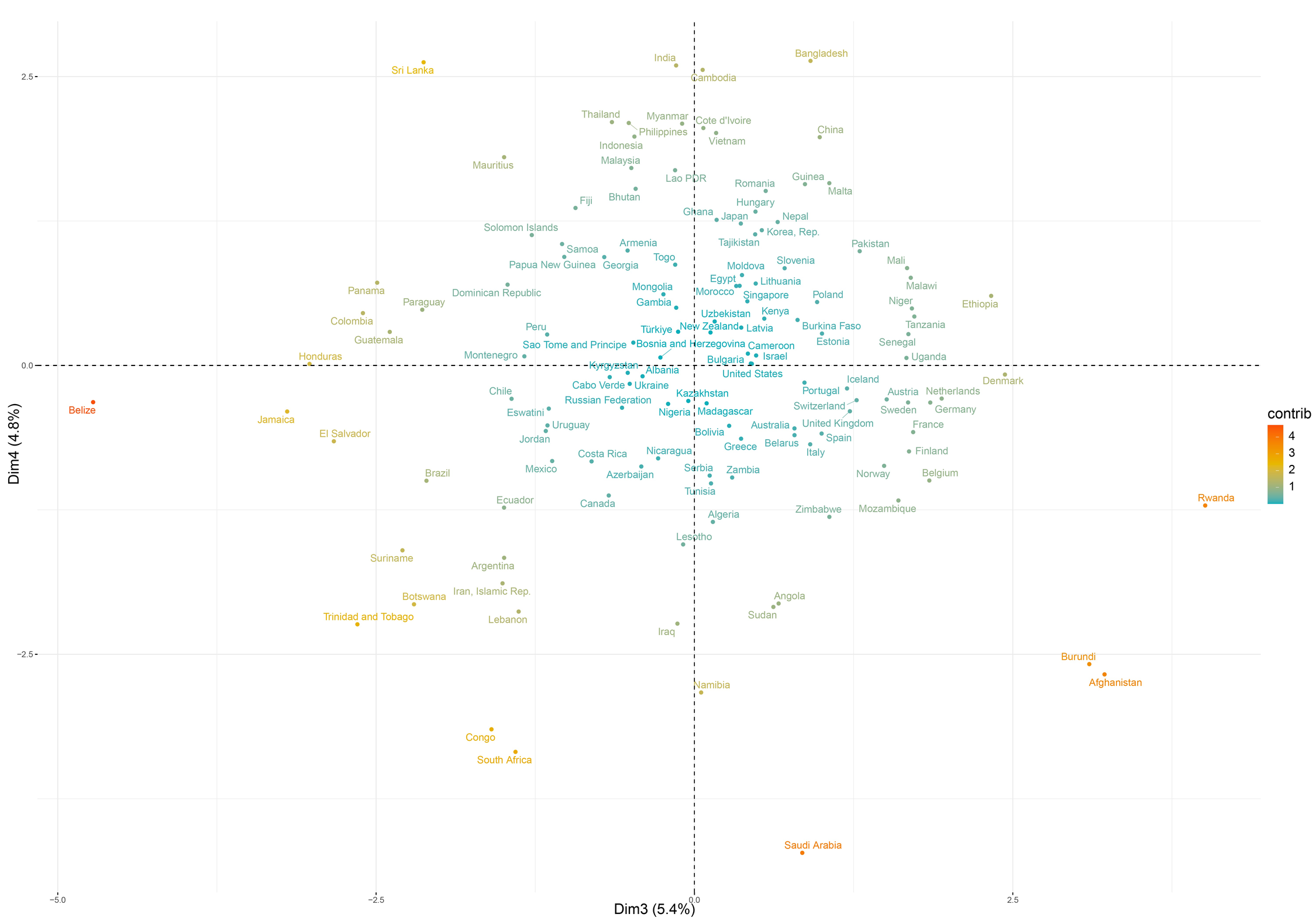

Supplement: S2 Fig — (TIF) [file pone.0310089.s006.tif]
