# Supplementary material for: What do the sustainable development goals reveal, and are they sufficient for sustainable development?
Source: PLoS One. 2024 Nov 4;19(11):e0310089. doi: 10.1371/journal.pone.0310089 (PMC11534252; doi:10.1371/journal.pone.0310089)
Supplement: Attachment — Submitted filename: Response to Reviewers.docx [file pone.0310089.s007.docx]

Thank you for all comments from the academic editor and reviewers, and they are of great help in improving our paper. Followings are our response to each point.

**Response to academic editor**

Thank you for your valuable comments on our paper. We have made modifications according to each of them and explained in detail in the following points.

**Point 1:** Please ensure that your manuscript meets PLOS ONE's style requirements, including those for file naming. The PLOS ONE style templates can be found at

<https://journals.plos.org/plosone/s/file?id=wjVg/PLOSOne_formatting_sample_main_body.pdf> and

<https://journals.plos.org/plosone/s/file?id=ba62/PLOSOne_formatting_sample_title_authors_affiliations.pdf>

Thank you for your valuable comment. Based on your provided PLOS ONE style templates, we have made several modifications.

First, we have corrected all level 1 and 2 headings in bold type, with 18pt and 16pt fonts, respectively.

Second, titles and citations of all figures and tables in the text and file naming for figures have been corrected, such as the “Fig 1”, “Fig 2”, etc.

Third, manuscript has been revised to double-space paragraph format.

Fourth, all reference citations have been revised as [1], [2], etc., and reference list has been put in order accordingly.

Fifth, supporting information citations have been revised in both in-text citations and the section after the reference list, such as “S1 Fig”, “S1 Table”, etc.

**Point 2:** We note that the grant information you provided in the ‘Funding Information’ and ‘Financial Disclosure’ sections do not match.

When you resubmit, please ensure that you provide the correct grant numbers for the awards you received for your study in the ‘Funding Information’ section.

Thank you for your valuable comment. We will check the “funding information” section carefully when resubmitting the revised manuscript.

**Point 3:** Thank you for stating the following financial disclosure:

[The paper has been supported by the Key Projects of the National Social Science Fundation (23AZD013).].

Please state what role the funders took in the study. If the funders had no role, please state: ""The funders had no role in study design, data collection and analysis, decision to publish, or preparation of the manuscript.""

If this statement is not correct you must amend it as needed.

Please include this amended Role of Funder statement in your cover letter; we will change the online submission form on your behalf.

Thank you for your valuable comment. We have included this amended Role of Funder statement in our cover letter, express as follows: “The funders had no role in study design, data collection and analysis, decision to publish, or preparation of the manuscript.”

**Point 4:** We note that you have referenced (ie. Husson, F., et al. []) which has currently not yet been accepted for publication. Please remove this from your References and amend this to state in the body of your manuscript: (ie “Husson, F., et al. [Unpublished]”) as detailed online in our guide for authors

<http://journals.plos.org/plosone/s/submission-guidelines#loc-reference-style>

Thank you for your valuable comment on our citations and references. In terms of this issue, we have removed this reference from body of the article and the reference list because the context we cited was also explained in another published article (Kassambara, 2017). Therefore, we delete the unpublished one (Husson, et al., 2010) and keep the published one (Kassambara, 2017).

**Point 5:** We are unable to open your Supporting Information file [Fig.rar]. Please kindly revise as necessary and re-upload.

Thank you for your valuable comment. We are sorry that the uploaded [Fig.rar] were unable to open, which caused the confusion when reviewers read our paper due to the missing figures. We have re-uploaded them with high resolution and changed to another compressed format [Fig.zip].

**Point 6:** We notice that your figures are uploaded with the file type 'Supporting Information'. Please amend the file type to 'figure'. Please ensure that each Supporting Information file has a legend listed in the manuscript after the references list.

Thank you for your comment. When we re-upload our revised version, we will amend the file type to ‘figure’. Besides, we have revised the supporting information section in the manuscript according to the PLOS ONE formatting sample, and all supporting information files have titles and legends listed after the references list, such as the “S1 Table”, “List of selected SDGs and illustrative groups”, “S1 Fig. Pairwise correlations among IG 1-4”, etc.

**Point 7:** Please include captions for your Supporting Information files at the end of your manuscript, and update any in-text citations to match accordingly. Please see our Supporting Information guidelines for more information: <http://journals.plos.org/plosone/s/supporting-information>.

Thank you for your valuable comment. We have revised captions, titles and in-text citations of supporting information.

As for the Supporting Information Section after the reference list, we have revised the expressions as “S1 Appendix”, “S1 Table”, “S2 Table”, “S1 Fig”, etc. In terms of those in-text citations, we have also revised them to match supporting information accordingly, such as the “…screening steps required for our statistical study (S1 Appendix), “…retained 133 countries (S3 Table) and 78 indicators…”, etc.

**Response to reviewer 1**

Thank you for your comments of merits on introduction, literature review and methodology in our paper. As for some minor comments suggested, we have made several modifications and explained in following two points in detail.

**Point 1:** - perhaps the research questions advanced in the first section could with advantage be relocated to the methodological section.

Thank you for your comment. As for our research, two research questions located in the Introduction Section could better highlight the purpose in the very beginning of the study. However, in the Methodological Section, we indeed neglected the mention of these two research questions. Therefore, in the revised version, a new paragraph has been added to relate the methodology with the two research questions mentioned in the Introduction Section, as shown below:

In our study, Multiple Factor Analysis (MFA) and Hierarchical Clustering on Principal Components (HCPC) were employed as primary tools in investigating the two research questions mentioned before. That is, what do the SDGs reveal, and are they sufficient to support a sustainable future?

**Point 2:** - some improvement in the last section could also be useful, namely providing more critical orientations for future research as well as for practical purposes.

Thank you for your comment. In our previous version, our limitations and orientations for future research were presented in the Conclusion Section in three aspects.

First, in the second paragraph from bottom, we summarized the third finding (sub-section 5.3) and proposed that “governance capacity constitutes the third PC and correlates with indicators covering all three pillars of sustainability, serving as a critical enabler of the SDGs, particularly the capability to prioritize different PVs in a timely and strategic manner with national condition changes”, indicating that the governance capacity that prioritizing different PVs timely and strategically is vital for sustainable development **for practical purpose.**

Second, in terms of the fourth finding (sub-section 5.4), we summarized as “neither EFP nor HPI could reveal the entire story of human development because they overlooked other PVs. However, they provide information that differs from the SDGs. Embracing more such indicators could enrich the theoretical and value bases of development, avoid development failures, and achieve a sustainable future”. We suggested that embracing more distinctive indicators that have low synergy with the GDP-similar indicators could enrich the value bases of development and achieve a sustainable future, which serving **for theoretical purpose.**

Third, we put forward two limitations in the last paragraph, including insufficiently regularly produced data and the limited selection of PCs. Whereas, in many cases, limitations could be converted to potential orientations for future research, and we neglected this concern in the previous version. **Therefore, regarding the two primary limitations, we have added their corresponding orientations.** Specifically, as for the first limitation, we suggest that considering that there are only five more years before 2030, it is of great urgency and significance to accelerate the collection of data and ensure the data quality. In terms of the second limitation, we indicate that the remaining PCs may still have specific meanings that are difficult to define and interpret, which could be a potential orientation for future research.

Overall, after the above modifications, we have proposed **orientations for future research in theoretical and practical aspects**, as well as **two orientations regarding two primary limitations**. We believe that these modifications could allow our paper to become more rigorous and practical.

**Response to reviewer 2**

Thank you for your comments on our paper, and they are constructive and helpful in improving our research. We have made several modifications according to each comment, are they are presented below:

**Point 1:** The indicators selected in the article are mainly related to economy and development, so it is not surprising to draw relevant conclusions.

Thank you for your valuable comment.

First, due to the **very limited data availability,** we have established rigorous criteria and screening steps to select indicators and countries (see S1 Appendix). For instance, indicators should cover sufficient countries rather than those with sufficient data in only a few countries, or indicators that can be compared globally were selected rather than those just for a subset of specific regions or categories of countries. For example, indicators in SDG 14 (Life Below Water) and other indicators related to oceans, inner lakes and mountains are not considered. Under such circumstances, we select indicators as much as possible to conduct statistical analysis, comprising 133 countries and 78 indicators after the data pre-processing (see S1 Table & S3 Table).

Second, among the selected 78 indicators, **the HDI and GDPP constitute the first principal component (PC) of the SDGs and determine country clusters.** In our study, we confirmed that despite the “beyond GDP” principle, economic values represented by GDP remain significant in propelling human development, and this explains why “GDP fetishism” is still prevalent nowadays.

Third, in our study, despite the result that the GDP per capita and HDI constitute the first PC (economic development dimension), the first PC only explain 27.9% of the SDGs, which explains that **SDGs comprises other dimensions of public values,** such as the second PC (inequality and environmental dimensions), third PC (governance capacity dimension) and fourth PC (economic growth rate). Specifically, in our first two research results, we confirm the contradiction that an increase in income level and HDI is achieved at the expense of high CO2 emissions per capita and higher EFP, with no significant improvements in ecological well-being represented by the HPI. Besides, the first two PCs approximately correspond to a combined Kuznets curve and an environmental Kuznets curve (EKC), which confirms the trade-offs between socioeconomic and environmental goals within the SDGs. For future sustainable development, SDGs must minimize such trade-offs and proceed within Earth’s carrying capacity. In our third research result (sub-section 5.3), we proposed that governance capacity constitutes the third PC and correlates with indicators covering all three pillars of sustainability, serving as a critical enabler of the SDGs, particularly the capability to prioritize different PVs in a timely and strategic manner with national condition changes. Our fourth research result (sub-section 5.4) presents that neither EFP nor HPI could tell the entire story of human development because they overlooked other PVs. However, they provide information that differs from the SDGs. Embracing more such indicators (those with low similarity with GDP) could enrich the theoretical and value bases of development, avoid development failures, and achieve a sustainable future.

To summary, on the one hand, **our selected indicators are not all related to economic development,** which were chosen after the rigorous criteria and screening steps. On the other hand, we not only discuss that GDP remain significant in propelling human development, **we also analyzed and discussed the later three PCs,** drawing conclusions like “prioritize different PVs in a timely and strategic manner with national condition changes” and “embracing more indicators like EFP and HPI to enrich the theoretical and public value bases of the SDGs to expand its dimensions for a better assessment and guidance of the global sustainable development.”

**Point 2:** There have been many empirical studies on Kuznets curve and environmental Kuznets curve in disciplines such as economics, this article only verifies the relevant conclusions.

Thank you for your valuable comment.

Indeed, in economics, environmental science and other disciplines, there are quite a few studies trying to verify the rationality of the Kuznets curve and the environmental Kuznets curve (EKC) from different perspectives and different scales. However, **in our study, the verification of both is to support the conclusions of this paper and lead to relevant research directions.**

Specifically, the first PC represents economic development dimension, while the second one is defined as the inequality and environmental dimensions. These two PCs correspond to the relationships between income level and inequality & environment. The projections of countries in the MFA’s first plane approximately correspond to a combined Kuznets curve and an environmental Kuznets curve (EKC) (see Figure 4). Therefore, our study attempts to discuss these two hypotheses to seek for practical suggestions for future sustainable development. In Figure 4, the countries in Cluster 1 were classified as having low income and human development levels, mainly from Sub-Saharan Africa and Southern Asia. On average, they performed better in terms of income equality and environmental quality. However, their good performance may be ascribed to their abundant natural resources, wide-spread poverty, and low levels of industrialization. Cluster 2 comprised the developing countries from Western Asia (especially the Gulf countries), Central Asia, Southeast Asia, Latin America, and the Caribbean with medium income and human development levels but a wide vertical distribution and worse performance in reducing inequality and environmental protection than Cluster 1. Finally, the countries in Cluster 3, primarily from North America and Europe, have the most stable and outstanding performance in achieving high income, controlling inequality, and protecting the environment.

Therefore, the nexus between economic growth, income inequality, and the environment raises an interesting but serious question: Can reducing inequality, environmental sustainability, and economic growth coexist in a nation’s early development phase? We take China’s development path as an example and considers such as development path is still difficult to reach, but worth studying and pursuing in the future. In our third research result (sub-section 5.3), we believe that governance capacity serves as a critical enabler of the SDGs, particularly the capability to prioritize different PVs in a timely and strategic manner with national condition changes, which is the key to achieve sustainable development.

To summary, the investigation of the Kuznets curve and environmental Kuznets curve (EKC) constitutes our second research result. That is, the projections of countries in the MFA’s first plane approximately correspond to a combined Kuznets curve and an environmental Kuznets curve (EKC). The question of whether economic growth can coexist with environmental sustainability and equality in the early stages of development is a meaningful topic for future research. This is an exploration of how to balance economic growth, environmental sustainability and social equity for sustainable development. **Its purpose is not only to verify the two hypotheses, but more importantly, to propose strategies for the two hypotheses for sustainable development, that is, to timely adjust the priority of different dimensions of public value based on the public value theory.**

**Point 3:** SDGs have been proposed for nearly a decade, so it is certain to have some lag.

Thank you for your valuable comment.

SDGs were proposed in 2015 and nearly a decade after its release. However, according to official reports, due to the huge uncertainty nowadays, SDGs are facing severe challenge and could not be achieved in 2030. **In the last decade, there are very few countries which could achieve sustainable development or achieve the SDGs. Therefore, despite some lag that SDGs have, the current situation indicates the urgency to accelerate the realization of the SDGs and strive for future long-term sustainable development for countries worldwide.**

**Given this, we explore the dimensions of sustainable development based on the public value theory, discovering realization path for SDGs.** Based on our research, we proposed several critical policy suggestions. We select two of them as examples here. First, governance capacity is a critical enabler of SDGs. Governments should strengthen cooperation across sectors and form holistic strategies to prioritize public values (PV) to avoid development failures. China’s logic of development presents as the creating of tremendous economic value in the early phases, then concentrating on other PV dimensions and achieve a more balanced and sustainable development. Second, neither EFP nor HPI are comprehensive, considering that human development is based on multidimensional PVs. What matters is to “jump out of the box,” exploring and embracing new theories, values, and indicators, which enables us to avoid development failures and achieve a sustainable future.

**Point 4:** In summary, the article lacks significant innovation and is not recommended for adoption.

Thank you for your valuable comments.

Our paper attempts to employ MFA and HCPC as primary tools and adopts **public value theory as theoretical foundation** to explore two research questions: 1) What are the main dimensions, objectives, and indicators of the SDGs, and how do they relate to each other? 2) Are SDGs sufficient to support a sustainable future?

We believe our research have several innovations.

First, **no study has placed SDGs under the theory of public value.** In our opinion, public value theory is the key theory that could enrich the dimensions of human development, guide the future sustainable development and avoid development failures. Specifically, the balance of different public value dimensions timely and strategically, as well as the exploring of new indicators, values and theories are critical for achieving a sustainable future. Therefore, the employ of the public value theory is our primary innovation point.

Secondly, SDGs has been expanding, their scale and structure has become more and more huge and complex. Hence, **it is necessary to assess its sufficiency and effectiveness in guiding global sustainable development.** However, there are not many studies concentrate on this topic. Therefore, we believe our research is necessary and innovative.

Thirdly, based on our research, we concluded four primary results (sub-sections 5.1-5.4), and several suggestions have been proposed for achieving global sustainable development. For example, governance capacity is a critical enabler of SDGs. Governments should strengthen cooperation across sectors and form holistic strategies to prioritize public values (PV) to avoid development failures. For another instance, neither EFP nor HPI are comprehensive, considering that human development is based on multidimensional PVs. What matters is to “jump out of the box,” exploring and embracing new theories, values, and indicators, which enables us to avoid development failures and achieve a sustainable future. Hence, we believe our study are of significance and practicability for avoiding development failure and achieving a sustainable future.

**Response to reviewer 3**

Thank you for your detailed and constructive suggestions on our paper, which are of great help in improving our research. As for each comment, we have made modifications or provided explanations, detailed are presented in the following points:

Objectives and Research Questions:

**Point 1:** The objectives of the paper are not fully justified and supported by the results. In the discussion section, objectives should connect the findings and discuss their contributions to addressing the research questions.

Thank you for your valuable comment. We will explain the relationships between research questions and findings of our research in two aspects.

**First, Q1: What are the main dimensions, objectives, and indicators of the SDGs, and how do they relate to each other?**

In our paper, sub-sections 5.1, 5.2 and 5.3 answer this question. Specifically, based on the SDGs and their indicators that contribute most to each PC, the first four PCs are defined as the economic development dimension, inequality and environmental dimensions, governance capacity dimension and economic growth rate dimension, respectively. Besides, in the sub-sections 5.1 and 5.4, we employed correlation circles (Fig 3) to investigate the relationships among indicators. For example, the circle constituted by the first two PCs displays that HDI and GDPP were well represented by the first PC and formed sharp angles with most other indicators. 13.2.2 (CO_2_ emissions per capita) contributed the most to the negative axis of the first PC, forming a sharp angle with EFP (its correlation value with the first PC was -0.78, S2 Table), suggesting that CO_2_ emissions per capita and EFP share similar information. Moreover, in the sub-sections 5.1 and 5.4, we employed “projections of countries on the MFA’s first (second) plane” (Fig 4, S2 Fig) to demonstrate the relationships between countries (observations) and PCs. For instance, as the Fig 4 presents, from left to right, the GDPP and HDI increased, indicating that the level of economic development is crucial for differentiating country clusters.

**Second, Q2: Are SDGs sufficient to support a sustainable future?**

In our paper, sub-section 5.4 answers this question. Specifically, among our 4 illustrative indicators (GDPP, HDI, EFP, HPI), even though they are also not comprehensive and exist some problems (please see the third and fourth paragraphs of the sub-section 5.4), EFP and HPI present their novelty in revealing flaws of the SDGs. EFP suggests that the current economy is propelled by the overconsumption of non-renewable natural resources and reveals the unsustainable aspects of the SDGs. HPI provides a distinctive perspective that focuses on environmental pressure-based well-being that the SDGs have not fully captured, namely, increasing well-being through an environmentally sustainable and socially just approach. Therefore, we suggest that the SDGs should embrace more indicators like EFP and HPI (with low correlations with GDP), which could enrich the theoretical and value bases of the SDGs for guiding global sustainable development in a comprehensive and practical way.

**Point 2:** Clearly state the objectives at the outset and ensure they align with the research questions and methodology. Explain how each objective was intended to contribute to the overall aim of the study.

Thank you for your valuable comment. **Our study proposed two research questions in the Introduction Section:**

Q1: What are the main dimensions, objectives, and indicators of the SDGs, and how do they relate to each other?

Q2: Are SDGs sufficient to support a sustainable future?

**We add a new paragraph at the beginning of the Methodology Section,** express as follows: In our study, Multiple Factor Analysis (MFA) and Hierarchical Clustering on Principal Components (HCPC) were employed as primary tools in investigating the two research questions mentioned before. That is, what do the SDGs reveal, and are they sufficient to support a sustainable future? **This allows the alignment of the research questions and methodology.**

In addition, our paper generated four results, respectively correspond to sub-sections 4.1, 4.2, 4.3, 4.4. **Among them, 4.1, 4.2 and 4.3 answer the first research question (Q1) while 4.4 answer the second research question (Q2).**

Specifically, the definitions of first four dimensions based on SDGs and indicators that contribute most are key step to answer our first research question (Q1: What are the main dimension, objectives, and indicators of the SDGs, and how do they relate to each other?). Besides, we select GDPP, HDI, EFP and HPI as illustrative indicators to answer the second research question (Q2: Are SDGs sufficient to support a sustainable future?). That is, the economic growth is still play a key role in the SDGs. For one thing, economic value is critical in driving other dimensions of human development. For another, SDGs require embracing more indicators like EFP and HPI to enrich the enrich the theoretical and value bases of development, avoid development failures, and achieve a sustainable future.

**Point 3:** In the discussion section, explicitly connect each finding to the stated objectives. Discuss how the results contribute to answering the research questions and whether any unexpected findings warrant further investigation or reinterpretation of the objectives.

Thank you for your valuable comment. We will explain this point and modifications in the following two aspects.

**First, how the result contribute the answering the research questions.**

Based on our Results and Discussion Section, we defined the first four PCs based on the SDGs and indicators with the highest contributions, respectively.

The definitions of first four dimensions are key step to answer our first research question (Q1: What are the main dimensions, objectives, and indicators of the SDGs, and how do they relate to each other?). They are finally defined as the economic development dimension (sub-section 5.1), inequality and environmental dimensions (sub-section 5.2), governance capacity dimension (sub-section 5.3) and economic growth rate dimension (sub-section 5.3), respectively.

The first three results (sub-section 5.1-5.3) answer the Q1. First, the HDI and GDPP are closely related to the SDGs and determine country clusters (5.1). Despite the “beyond GDP” principle, economic values represented by GDP remain significant in propelling human development, and this explains why “GDP fetishism” is still prevalent nowadays. Our study confirms the contradiction that an increase in income level and HDI is achieved at the expense of high CO2 emissions per capita and higher EFP, with no significant improvements in ecological well-being represented by the HPI. For future sustainable development, SDGs must minimize trade-offs between socioeconomic and environmental goals and proceed within Earth’s carrying capacity. Second, the projections of countries in the MFA’s first plane approximately correspond to a combined Kuznets curve and an environmental Kuznets curve (EKC) (5.2). The question of whether economic growth can coexist with environmental sustainability and equality in the early stages of development is a meaningful topic for future research. Third, governance capacity constitutes the third PC and correlates with indicators covering all three pillars of sustainability, serving as a critical enabler of the SDGs, particularly the capability to prioritize different PVs in a timely and strategic manner with national condition changes (5.3). Notably, some countries suffer from the growth slowdowns from Latin America and Southeast Asia have fallen into the “middle-income trap”, is mainly caused by weak governance capacity.

Besides, we select GDPP, HDI, EFP and HPI as illustrative indicators to answer the second research question (Q2: Are SDGs sufficient to support a sustainable future?). That is, the economic growth is still play a key role in the SDGs. For one thing, economic value is critical in driving other dimensions of human development. For another, SDGs are still not comprehensive, they require embracing more indicators like EFP and HPI to enrich the enrich the theoretical and value bases of development to better guide the global sustainable development.

**Second, whether any unexpected findings warrant further investigation or reinterpretation of the objectives.**

In our paper, one significant finding that worth future investigating is the selection and definition of PCs. Although 78 PCs were generated from the MFA, we could only select the top four PCs with the most considerable variances because the remaining PCs were not easy to define and interpret, which may be attributed to large data noise derived from data screening and missing data imputation. However, these remaining PCs may still have meaningful definitions, which could be a potential orientation for future research. This point has been explained in detail in the last paragraph of the Conclusion Section.

**Point 4:** Identify any gaps or limitations in achieving the objectives based on the findings and propose future research directions to address these gaps.

Thank you for your valuable comment. Our expression of limitations in the previous version is not clear and organized. Therefore, **we have made some modifications, clearly listed two limitations, and proposed future research directions to address these gaps.** We have revised the last paragraph of the Conclusion Section as follows:

Finally, our study has two primary limitations. First, the SDGs remain incompletely measured, and this situation has deteriorated further owing to the COVID-19 pandemic and regional conflicts. Considering that there are only five more years be-fore 2030, it is of great urgency and significance to accelerate the collection of data and ensure the data quality. Insufficiently regularly produced data from many countries (especially sub-Saharan African countries) limited our study to a cross-sectional analysis. Using a subset of indicators may have some potential impacts on the result. However, we believe such impacts would hardly influence our findings and policy suggestions because most indicators from SDGs have medium to high correlations with GDPP or HDI. With more data becoming available in the future, statistical analyses should be repeated to increase the robustness of our research. Another limitation was the selection and definition of PCs. Although 78 PCs were generated from the MFA, we could only select the top four PCs with the most considerable variances be-cause the remaining PCs were not easy to define and interpret, which may be attributed to large data noise derived from data screening and missing data imputation. However, remaining PCs may still have specific meanings that are difficult to define and interpret, which could be a potential orientation for future research.

Methodology:

**Point 5:** Ambiguity in the selection of SDGs and their indicators, exclusion of SDG 14, and the limited number of indicators used.

Thank you for your valuable comment. Our paper adopted rigorous criteria and screening steps to select indicators and countries, and details are presented in S1 Appendix. Under these criteria, we try to choose as many indicators as possible.

As for the indicator selection, we followed the criteria below:

1.	Indicators should cover sufficient countries rather than those with sufficient data in only a few countries (i.e., developed countries).

2.	Indicators should be expressed in the form of continuous numerical variables. Di-chotomous variables (0 or 1) and ordinal variables (1-5: unsatisfied to satisfied) are excluded.

3.	Total indicators rather than sub-indicators (i.e., those classified by sex and loca-tion) were chosen as far as possible.

4.	Indicators that can be compared globally were selected rather than those just for a subset of specific regions or categories of countries. For example, indicators in SDG 14 (Life Below Water) and other indicators related to oceans, inner lakes and mountains are not considered.

5.	Ratio, rate, or per capita indicators are selected as far as possible to minimize the impact of the size and volume of the country, such as 12.2.2 (domestic material consumption per GDP), 13.2.2 (CO2 emissions metric tons per capita), IG 3 (ecological footprint per capita).

6.	As for the real GDP growth rate indicators (8.1.1 and 8.2.1), arithmetic means between 2015 and 2019 (5 years) were adopted to reduce the impact of the ab-normal value of the single year (2019) on the statistical results.

7.	On the premise of using indicators from the official database of the United Na-tions as many as possible, this study replaced a few indicators (i.e., 2.2.1, 4.2.2, 8.5.2, 11.1.1) that do not meet the first criteria with similar ones from the World Bank WDI database. Generally, the proportion of indicators from the UN Global SDG database reached 87%.

8.	Illustrative groups (IGs) 1-4 have not participated in the MFA and HCPC. IG 1 contains GDPP (GDP per capita), IG 2 includes HDI, IG 3 comprises EPI, and IG 4 is made up of HPI. Significantly, these four groups were selected for comparison purposes with the SDGs.

After the above selection, 215 countries (and regions) and 95 indicators (excluding four illustrative indicators) are screened. Then, the remaining indicators and countries underwent the second screening, followed by the missing data imputation. Criteria and steps are presented below:

1.	For indicators with no 2019 data, the data of the nearest year were used instead (1.3.1, 3.1.1, 3.d.1, 5.2.1, 6.5.1, 8.10.2, 11.1.1, 16.7.1, EFP).

2.	Based on the first step, delete countries with a proportion of missing values great-er than 30%, leaving 164 countries.

3.	Based on the second step, delete indicators with a proportion of missing values greater than 30%, leaving 78 indicators.

4.	Based on the third step, we filled in missing values via the classic LOCF (Last observation forward) method with the most recent data from 2014 to 2018 (within five years).

5.	Country samples whose missing values were still greater than 7% after filling have been deleted. Regarding remaining samples, we further filled in their miss-ing values by searching data from other official databases (i.e., UNESCO), inter-national economic and political organizations (i.e., OECD, EU), UN reports, and the mean values of region or income.

Finally, we managed to keep 133 countries (S3 Table) and 78 indicators (exclude four illustrative indicators) (S1 Table) that were proceeded for MFA and HCPC.

**Point 6:** Provide a rationale for why specific SDGs and indicators were chosen over others. Consider the relevance to the research questions, data availability, and the ability to provide a comprehensive analysis.

Thank you for your valuable comment. I will explain this point in the following 3 aspects.

**First, data availability.** As mentioned in Point 5, after rigorous selection criteria and screening steps, we finally keep 133 countries (S3 Table) and 78 indicators (exclude four illustrative indicators) (S1 Table) for later statistical analysis. Our purpose is to keep as many as indicators in the analysis as possible, and we think 133 countries and 78 indicators (cover 16 SDGs, exclude SDG 14, reason mentioned in Point 5) are sufficient in this research because they are mainly representative indicators.

**Second, relevance to the research questions.** In our research, we proposed two research questions:

Q1: What are the main dimensions, objectives, and indicators of the SDGs, and how do they relate to each other?

Q2: Are SDGs sufficient to support a sustainable future?

As for the screened 78 indicators, they are used as initial indicators for answering the Q1. That is, to determine the main dimensions, objectives, and indicators of the SDGs, and investigate how do they relate to each other.

In terms of the 4 illustrative indicators (GDPP, HDI, EFP, HPI), they are served as indicators to answer the Q2. On one hand, based on our statistical analysis and findings, GDPP and HDI relate closely to SDGs, notably, HDI could explain 94% of the first PC. Besides, GDPP and HDI are surrounded by most indicators from SDGs, suggesting the significance of economic development in SDGs. On the other hand, EFP and HPI are employed to support the contradiction that an increase in income level and HDI is achieved at the expense of high CO_2_ emissions per capita and higher EFP, with no significant improvements in ecological well-being represented by the HPI. Therefore, we suggest embracing more such indicators to enrich the theoretical and value bases of development, avoid development failures, and achieve a sustainable future.

**Third, the ability to provide a comprehensive analysis.** Based on the 78 representative indicators and 4 illustrative indicators selected, we believe our research could be able to provide a relatively comprehensive analysis as far as possible.

**Point 7:** Acknowledge the limitation of using a subset of indicators and justify how these selected indicators are representative of the broader dataset. Discuss the potential implications of excluding certain indicators.

Thank you for your valuable comment. I will explain this question in two aspects.

First, as mentioned in the Point 5 & 6, we try to select representative indicators from the SDGs as many as possible. Details about the selection criteria and screening steps, please see S1 Appendix or Point 5.

Second, indeed, using a subset of indicators could have some potential impacts on the result. However, we believe that the results will not change a lot with more other indicators revolved because majority of indicators from SDGs have medium-to-high correlations with GDPP or HDI. However, to increase the robustness and rigor of the research, limitation of using a subset of indicators should be explained in the final section (Conclusion Section). Therefore, in the Conclusion Section, we have made some modifications in the final paragraph. Specifically, in terms of the first limitation (data availability limitation), we express as follows, “First, the SDGs remain incompletely measured, and this situation has deteriorated further owing to the COVID-19 pandemic and regional conflicts. Considering that there are only five more years before 2030, it is of great urgency and significance to accelerate the collection of data and ensure the data quality. Insufficiently regularly produced data from many countries (especially sub-Saharan African countries) limited our study to a cross-sectional analysis. Using a subset of indicators may have some potential impacts on the result. However, we believe such impacts would hardly influence our findings and policy suggestions because most indicators from SDGs have medium to high correlations with GDPP or HDI. With more data becoming available in the future, statistical analyses should be repeated to increase the robustness of our research.”

**Point 8:** Consider categorizing countries based on multiple criteria, including ecological footprint (EFP) and Happy Planet Index (HPI), in addition to Gross Domestic Product per capita (GDPPC) and HDI. Provide a rationale for the chosen criteria and discuss how these categories align with the research objectives.

Thank you for your valuable comment. We will explain this point in three aspects.

**First, the clustering criteria of HCPC.** We categorized countries using HCPC (hierarchical clustering on principal components), and steps or principles could be referred in sub-section 4.2. Based on the HCPC, 133 countries have been classified as 3 categories (38, 58 and 37 countries). Specifically, in our study, there are 78 indicators from the SDGs (involved in HCPC) and 4 illustrative indicators (not involved in HCPC). HCPC is conducted based on the first for principal components (PCs) derived from 78 indicators, not based on 4 illustrative indicators (GDPP, HDI, EFP, HPI).

**Second, the categorization based on multiple criteria.** According to Fig 4, in the axis constituted by first two PCs, from left to right, the GDPP and HDI increased, indicating that the level of economic development is crucial for differentiating country clusters. Hence, HDI and GDPP relate closely to SDGs. However, if we category countries according to the EFP or HPI, the results may be different because the rankings of this two differ from most other indicators, which providing some enlightening information about the sustainable development. Your suggestion of categorizing countries based on multiple criteria is meaningful and instructive. However, due to the length limit, this could be an interesting and promising research in the future, thank you!

**Third, how these categories align with the research objectives.** Clustering 133 countries into 3 clusters is aimed at investigating critical factor that differentiating countries based on the first four PCs. According to the Fig 4 and the second paragraph from bottom of sub-section 5.1, from left to right, the GDPP and HDI increased, indicating that the level of economic development is crucial for differentiating country clusters. This result verifies the finding that GDPP and HDI contribute most to the first PC and relate closely to SDGs. The result answers the two research questions (Q1 & Q2). Specifically, GDPP and HDI contributes most in the first PC and are critical in differentiating countries (Q1). Besides, despite the progress and comprehensiveness of the SDGs compared to most other indicators, they are still required to embrace more theories, dimensions and indicators to become more comprehensive and practical (Q2).

Results and Discussion:

**Point 9:** Confusions between suggested dimensions based on HCPC-selected indicators and the trade-off between socioeconomic and environmental goals. Figures are missing in paper.

Thank you for your valuable comment. We will explain this question in the following 3 points.

**First, our suggested dimensions (please see the following Fig 1).** Because 78 indicators were used as variables in the analysis, the MFA generated 78 PCs, but only those with high eigenvalues (usually higher than 1) were selected as PCs. In our study, the first eight PCs satisfied the criterion, and half exceeded 1.5. In addition, using the scree test method demonstrated in Fig 1, the first four PCs explained a total variance of 45.7% and were selected as the target PCs. In the Results and Discussion Section, these four PCs are defined as the economic development dimension, inequality and environmental dimensions, governance capacity dimension, economic growth rate dimension, respectively, and this section sequentially presents our findings based on these first four PCs.

**Fig 1. Scree plot**

**Second, HCPC (hierarchical clustering on principal components).** In our study, HCPC is employed to cluster 133 countries based on previous selected 4 principal components (PCs) (3 Clusters derived from HCPC are presented in S3 Table). The purpose is to identify critical factors that differentiating countries regarding performance on sustainable development. From left to right, the GDPP and HDI increased, indicating that the level of economic development is crucial for differentiating country clusters.

**Fig 4. Projections of countries on the MFA’s first plane**

**Third, trade-off between socioeconomic and environmental goals.** The trade-offs between socioeconomic and environmental goals are primarily displayed in the Fig 3 below. As demonstrated in the first circle constituted by the first two PCs, most indicators with high contributions densely surrounded the positive axis of the first PC, implying high similarities. HDI and GDPP were well represented by the first PC and formed sharp angles with most other indicators, demonstrating that most indicators synergize with a country’s economic development level. Significantly, 13.2.2 (CO2 emissions per capita) contributed the most to the negative axis of the first PC, forming a sharp angle with EFP (its correlation value with the first PC was -0.78, S2 Table), suggesting that CO2 emissions per capita and EFP share similar information. This result revealed that current global developments fail to constrain themselves within the Earth’s carrying capacity. Moreover, HPI was not significantly correlated with the first PC (0.33, S2 Table), indicating that an increase in GDPP or HDI does not always contribute to ecological well-being.

**Fig 3. Correlation circles with 30 indicators with the highest contributions**

To summary, the selected four PCs represent four specific dimensions of sustainable development and constitutes the four findings (sub-sections 5.1-5.4) in the Result and Discussion Section. HCPC is a useful tool for investigating key factors in differentiating country’s performance on sustainable development. Meanwhile, the trade-offs between socioeconomic and environmental goals are mainly presented in the first finding (sub-section 5.1: HDI and GDPP relate closely to SDGs), which aimed at indicating the fact that global development nowadays is achieved at the expense of high CO_2_ emissions and higher ecological footprint (EF). Due to some file upload mistakes, the academic editor told me that he is unable to open my uploaded figure files [Fig.rar], and that is the reason for the missing figures in our paper. To solve this problem, we have revised our figures with high resolution and re-uploaded [Fig.zip], we are sorry for this problem.

**Point 10:** Clarify how the selected dimensions align with the HCPC-selected indicators and why they are relevant to the research objectives. Discuss any trade-offs identified in the results and how they relate to achieving sustainable development goals.

Thank you for your valuable comment. This point will be clarified in two aspects.

**First, dimensions, indicators and their relevance to the research questions.**

Based on our Results and Discussion Section, we defined the first four PCs based on the contributions of SDGs and indicators with the highest contributions to the first four PCs, respectively. Take the second PC as example. The second PC explained 7.6% of the total variance, with SDG 10 (reduced inequalities), SDG 15 (life on land), SDG 6 (clean water and sanitation), SDG 5 (gender equality), and SDG 12 (responsible consumption and production) contributing the most (Table 2). SDGs 10 and 5 comprise indicators focusing on general inequality issues, including income and gender inequalities, such as 5.5.2 (proportion of women in managerial positions), 10.4.1 (labor share of GDP), 10.4.2 (Gini index) and 5.5.1 (proportion of seats held by women in national parliaments). SDGs 6, 15, and 12 include indicators regarding climate change, biodiversity loss, and pollution, such as 6.4.2 (level of water stress, freshwater withdrawal as a proportion of available fresh-water resources), 12.c.1 (Fossil-fuel subsidies as a proportion of total GDP), 15.1.1 (forest area as a proportion of total land area), and 15.1.2 (average proportion of terrestrial key biodiversity areas (KBAs) covered by protected areas (%)). Moreover, most other SDGs that contribute considerably to the second PC, including SDGs 7, 9, and 11, and their indicators that appear in the top 20 contributors to the second PC, also measure inequality or the environment (Table 3), such as 11.6.2 (annual mean levels of fine particulate matter), 9.4.1 (carbon dioxide emissions per unit of manufacturing value added) and 7.2.1 (renewable energy share in the total final energy consumption). Therefore, the second PC could be defined as inequality and environmental dimensions.

The definition of first four dimensions and indicators are key step to answer our first research question (Q1: What are the main dimensions, objectives, and indicators of the SDGs, and how do they relate to each other?). Besides, we select GDPP, HDI, EFP and HPI as illustrative indicators to answer the second research question (Q2: Are SDGs sufficient to support a sustainable future?). That is, the economic growth is still play a key role in the SDGs. For one thing, economic value is critical in driving other dimensions of human development. For another, SDGs require embracing more indicators like EFP and HPI to enrich the enrich the theoretical and value bases of development, avoid development failures, and achieve a sustainable future.

**Second, trade-offs and how they relate to achieving SDGs.**

**The discussion of potential trade-offs and how they relate to achieving SDGs is one of the most crucial contents in our paper.** Trade-offs are explained primarily in the sub-sections 5.1 and 5.2.

In sub-section 5.1, as the Fig 3 shows, 13.2.2 (CO2 emissions per capita) contributed the most to the negative axis of the first PC, forming a sharp angle with EFP (its correlation value with the first PC was -0.78, S2 Table), suggesting that CO2 emissions per capita and EFP share similar information. This result revealed that current global developments fail to constrain themselves within the Earth’s carrying capacity. Moreover, HPI was not significantly correlated with the first PC (0.33, S2 Table), indicating that an increase in GDPP or HDI does not always contribute to ecological well-being.

In sub-section 5.2, considering that the first PC is the dimension of economic development, while the second PC represents the dimensions of inequality and the environment, notably, the countries in Fig 4 constitute a curve corresponding to a combined Kuznets curve and environmental Kuznets curve (EKC). In our case, the countries in Cluster 1 were classified as having low income and human development levels, mainly from Sub-Saharan Africa and Southern Asia. On average, they performed better in terms of income equality and environmental quality. However, their good performance may be ascribed to their abundant natural resources, widespread poverty, and low levels of industrialization. Cluster 2 comprised the developing countries from Western Asia (especially the Gulf countries), Central Asia, Southeast Asia, Latin America, and the Caribbean with medium income and human development levels but a wide vertical distribution and worse performance in reducing inequality and environmental protection than Cluster 1. Finally, the countries in Cluster 3, primarily from North America and Europe, have the most stable and outstanding performance in achieving high income, controlling inequality, and protecting the environment. Therefore, the nexus between economic growth, income inequality, and the environment raises an interesting but serious question: Can reducing inequality, environmental sustainability, and economic growth coexist in a nation’s early development phase?

Therefore, after identifying the trade-offs between socioeconomic goals and environmental goals and the earth’s capacity (EFP), we propose solutions to deal with such trade-offs in the sub-sections 5.3 and 5.4, especially taking China’s development experience as an example.

In sub-section 5.3, in the second paragraph from bottom, we discussed that “To reinforce synergies and minimize trade-offs for sustainable development, forming a holistic view across different PV dimensions and public sectors has become increasingly crucial and stringent, especially regarding the capability to prioritize different PVs in a timely and strategic manner and monitor relevant indicators effectively. Notably, a “Whole of Government” approach that emphasizes the active employ of formal and informal networks across different agencies within the government to increase integration, coordination, and capacity is increasingly critical for SDGs.” Then, we showed how China demonstrates the dynamic prioritization of public values (PVs) during its journey from lopsided development to a much more balanced development via integrated growth. The primary logic was to create tremendous economic value in the early phases, enabling China to concentrate on other PV dimensions and achieve sustainable development.

**In sub-section 5.4, we believe that trade-offs are not always the bad thing. Instead, in many cases, some trade-offs could reveal potential problems existed, and suggest new orientations for sustainable development.** In our paper, EFP and HPI provide us with enlightening information differ from GDPP or HDI and diagnoses some potential issues and overlooked PVs regarding SDGs. Specifically, EFP suggests that the current economy is propelled by the overconsumption of non-renewable natural resources and reveals the unsustainable aspects of the SDGs. HPI provides a distinctive perspective that focuses on environmental pressure-based well-being that the SDGs have not fully captured, namely, increasing well-being through an environmentally sustainable and socially just approach. In addition to EFP and HPI, there are many other similar indicators, we only use EFP and HPI as representatives. What the SDGs need to do is to “jump out of the box” and explore new theories, dimensions and indicators based on public values, which enhance the comprehensiveness of the SDGs and better guide the future sustainable development.

**Point 11:** Address the interlinkages between economic, social, and environmental sustainability beyond CO2 emissions, EFP, GDPPC, and HDI. Consider integrating additional indicators or dimensions to provide a more comprehensive analysis.

Thank you for your valuable comment.

In our paper, apart from 78 indicators from the SDGs, we selected 4 illustrative indicators (GDPP, HDI, EFP, HPI) to facilitate the data analysis and results interpretation. Notably, these 4 indicators are only for illustrative purposes and not involved in the statistical analysis including MFA and HCPC.

GDPP and HDI are used to demonstrate their contributions to the first PC, revealing that the economic value is still significant in the SDGs. EFP and HPI are adopted to test the sufficiency of the SDGs. They provide enlightening information that diagnoses some potential issues and overlooked PVs regrading SDGs. Details about these four illustrative indicators are explained in our Results and Discussion Section.

However, we only select EFP and HPI as representative indicators to demonstrate our ideas. In fact, there are many other indicators like these two which are differs from GDP or HDI. Due to the length limit, we could not list all other indicators like EFP or HPI, and this is not the primary purpose of our paper. Integrating additional indicators or dimensions to provide a more comprehensive analysis may be an interesting and promising orientation for future research.

We believe that neither EFP nor HPI is comprehensive. Development is not static. What matters for the SDGs is to embrace new theories, explore more dimension and indicators of sustainable development, which enables the SDGs to become more comprehensive and practical.

**Point 12:** Discuss GDPPC as a measure of economic growth and HDI as a measure of human development and well-being. Justify their relevance in assessing sustainable development outcomes and discuss any limitations in their application.

Thank you for your valuable comment. Your comment is just the idea we organized our paper.

One of the purposes of our paper is to explore the sustainable development and its dimensions, especially how to achieve sustainable development. I will explain ideas of our paper briefly in the following points.

First, GDP and GDPPC (GDP per capita) are invented in 1930s and have been regarded as one of the most prominent indicators for measuring a country’s development and was honored as the greatest invention of the 20^th^ century since the 1940s. However, the use of GDP as a proxy for human development has attracted widespread criticism. Not all social phenomena and human behaviors can be monetarized as a reference to market prices, such as social justice, individual mental health, and natural resources that money cannot fully measure. The economic way of thinking has earned a reputation for knowing the price of everything but the value of nothing. As many valuable items are excluded and materialized by GDP, we appear to be richer than we really are, while the reality is that we are losing many valuable values and resources. Therefore, a great number of indicators that attempted to improve, replace or supplement GDP appeared. Details about some of these representative indicators could be found in the sub-section (2.2) of our paper.

Second, HDI is the best-known alternative to GDP for measuring human development and well-being and is composed of income, health, and education dimensions. Although it has often been criticized for its incomplete assessment of human development and high correlation with GDP, it can still reveal many aspects that beyond GDP. However, like many other composite indicators, single indicator or index can not tell the whole story of the human development. Assessing a country’s development based on one or several single indicators are not comprehensive.

Third, a more comprehensive way of assessing human development is to adopt “indicator suites” that comprises many indicators allocated to different themes, such as the Miennium Development Goals (MDGs) and SDGs. However, even though these types of indicator suites are constituted by a huge number of indicators that successfully go beyond GDP, they are still not comprehensive. Hence, our paper is aimed at deconstructing SDGs and investigating whether they are sufficient to support a sustainable future based on the public value theory. Based on our research, despite that SDGs are much more comprehensive than any other indicator, majority of their indicators are still correlated with the GDP per capita (GDPP) or HDI. For one thing, it verifies that economic growth is crucial in human development and could drive most of the other dimensions. For another, it indicates that SDGs are expected to expand their dimensions, indicators (especially those with low correlations with GDP, such as EFP and HPI) based on public value theory, which enables us to enrich our understanding of sustainable development and avoid development failures.

To summary, **GDP, HDI and SDGs are products with the human’s understanding of development deepen.** Our paper is primarily concentrated on exploring the content of the current SDGs, investigating their sufficiency and seek for realization path for future sustainable development.

**Point 13:** By addressing these points in your paper, you can enhance the clarity, justification, and robustness of your research methodology, results interpretation, and discussion of findings. This approach will ensure that your paper effectively addresses the complexities of sustainability and provides valuable insights into the interplay between economic, social, and environmental factors.

Thank you for your valuable suggestions on our paper, which allow us to enhance the robustness of our methodology, better interpret results and effectively proposing policy suggestions.

In the last version, due to some file upload problems, our 6 figures were unable to open, which caused some confusion and misunderstanding of key research findings. To solve this problem, we have revised our figures and re-uploaded in high resolutions. We are sorry for this problem.
